# Unraveling the interconnectedness between physician burnout and symptoms of depression, anxiety, and stress: a network analysis among Chinese psychiatrists

**DOI:** 10.3389/fpubh.2024.1493424

**Published:** 2025-01-07

**Authors:** Song Wang, Mengyue Gu, Shujing Zhang, Jingyang Gu, Yudong Shi, Yating Yang, Ling Zhang, Mengdie Li, Lei Xia, Feng Jiang, Huanzhong Liu, Yi-lang Tang

**Affiliations:** ^1^Department of Psychiatry, Chaohu Hospital of Anhui Medical University, Hefei, China; ^2^Anhui Psychiatric Center, Hefei, China; ^3^Department of Psychiatry and Behavioral Sciences, Emory University, Atlanta, GA, United States; ^4^School of International and Public Affairs, Shanghai Jiao Tong University, Shanghai, China; ^5^Institute of Healthy Yangtze River Delta, Shanghai Jiao Tong University, Shanghai, China; ^6^Institute of Health Policy, Shanghai Jiao Tong University, Shanghai, China; ^7^Institute of Grand Health, Wenzhou Medical University, Wenzhou, China; ^8^Affiliated Psychological Hospital of Anhui Medical University, Hefei, China; ^9^Department of Psychiatry, School of Mental Health and Psychological Sciences, Anhui Medical University, Hefei, China; ^10^Atlanta VA Medical Center, Decatur, GA, United States

**Keywords:** physician burnout, stress, depression, anxiety, psychiatrists, network analysis

## Abstract

**Background:**

The COVID-19 pandemic significantly increased the levels of burnout and symptoms of depression, anxiety, and stress among healthcare professionals. However, research on the interrelations between burnout and psychological symptoms is scarce, particularly among psychiatrists. This study addresses this gap in a national sample.

**Method:**

Data was collected via an online survey conducted in Mainland China from January to March 2021 with a sample size of 3,783 participants. Psychological symptoms were assessed using the Depression, Anxiety, and Stress Scale-21 (DASS-21), and physician burnout was assessed using the Maslach Burnout Inventory-Human Service Survey (MBIHSS). Network analysis was used to examine the interconnection between physician burnout and psychological symptoms, with further analysis conducted on multiple levels, including individual symptoms in central positions or acting as bridges between clusters, and identifying core symptom combinations with significant correlations.

**Results:**

Stress emerged as the highest Expected Influence (EI) index, with emotional exhaustion in the burnout cluster being the singular bridge symptom. Furthermore, depressive symptoms such as hopelessness and anhedonia showed a strong and the most straightforward association with emotional exhaustion, while stress-related overreaction was closely associated with depersonalization.

**Conclusion:**

Network analysis between burnout and psychological symptoms identified critical symptoms like stress and emotional exhaustion in Chinese psychiatrists. Close monitoring of these symptoms may be crucial for mitigating the risk of common psychological disturbances and preventing their exacerbation in this population.

## 1 Introduction

Physicians, including psychiatrists, are particularly vulnerable to a range of psychological symptoms such as depression, anxiety, stress, and insomnia (1–3). The COVID-19 pandemic has exacerbated these issues due to an increased workload and other stressors. Facing this global public health crisis, medical professionals must deal with immense work pressure, a higher risk of infection, and continuous emotional and psychological stress. A recent nationwide survey conducted by Yao et al. (4) revealed that Chinese psychiatrists experienced high levels of burnout during the pandemic, highlighting the significant impact of these pressures on their job satisfaction and overall mental health.

In addition to the heavy workload, medical professionals also face social stigma. Social stigma refers to the negative perceptions and discriminatory attitudes directed towards healthcare workers, stemming from their close associations with infectious diseases (5). This stigma can lead to social ostracization and emotional distress, further compounding the challenges these professionals face (6). According to Malik and Annabi (7), such psychological pressures, combined with professional burnout, can have a lasting effect on the mental well-being of healthcare workers, suggesting that intervention is critical. Summers et al. (8) further emphasized the global scope of this issue, noting that psychiatrists in North America also experienced elevated levels of depression, anxiety, and burnout during the pandemic. Kang et al. (9) noted that medical professionals who directly managed COVID-19 cases and were exposed to high-pressure environments were more susceptible to mental health issues such as depression, anxiety, and stress.

The COVID-19 pandemic has underscored the critical need for effective mental health interventions (10). During this period, psychiatrists have played an essential role in providing mental health support while facing unique challenges and pressures (11, 12). There has been an unprecedented surge in demand for psychiatrists that significantly increase their workload and stress levels since the COVID-19 pandemic (13). Especially in China, the mental health support system is facing a dual challenge of resource scarcity and uneven distribution, making it difficult to meet the public’s growing demands for mental health services. According to government staffing guidelines, fewer than one-third of hospitals (31.7%) meet the minimum ratio of psychiatrists per bed, highlighting the insufficiency of resource allocation (14). They frequently manage complex cases involving severe psychological disorders and intense emotional fluctuations (15, 16), substantially increasing their work-related stress.

Moreover, widespread physician burnout, emotional exhaustion, and psychological distress underscore the challenges faced by medical professionals (17). Physician burnout is defined as a psychological syndrome stemming from enduring interpersonal stressors in medical practice (18). It is characterized by emotional exhaustion—described as a condition in which individuals feel emotionally overstretched and drained of emotional resources (19), depersonalization, and a diminished sense of personal achievement. Previous research predominantly viewed burnout as just another manifestation of depressive symptoms (20, 21). However, this perspective was challenged by Maslach and Leiter (22) in their study, suggesting that the link between burnout and depression might not be straightforward. Despite this re-evaluation, physician burnout remains to be linked to a broad spectrum of psychiatric conditions and risky behaviors. For instance, West et al. (23) revealed that doctors experiencing physician burnout were at an increased risk of emotional disorders, substance and alcohol use disorder, and motor vehicle accidents, illustrating the complex implications of physician burnout beyond its connection with depression.

A recent study increasingly demonstrated the intricate connections between depression, and anxiety across the three dimensions of physician burnout: emotional exhaustion, depersonalization, and reduced personal accomplishment (24). While these investigations provided a broad overview of the associations among various symptoms, they did not delve into any specific links. Network analysis (NA) techniques offer a novel approach to moving beyond the traditional categorical definitions of mental disorders, focusing on the interplay among individual symptoms rather than broader syndromes, thereby uncovering the underlying associations within psychopathology (25, 26). NA enables the modeling of interactions between psychological structures at the symptom level. This method employs “nodes” to represent various psychological symptoms and “edges” to depict the connections between them. Previous research often concentrated on the structural relationships between depression and anxiety (27), potentially overlooking the unique links between the three dimensions of burnout and various mental health symptoms. By leveraging NA, especially through the use of partial correlations and regularization techniques, we can unveil the subtle connections among physician burnout, depression, anxiety, and stress. Moreover, by calculating the bridge expected influence (BEI) index, which sums the edges connecting a node to nodes in other clusters, we can better understand the protective role of various physician burnout dimensions on mental health, providing fresh insights for potential intervention strategies.

Through the utilization of NA, this study aims to achieve two objectives: (1) examine the associations between physician burnout symptoms (emotional exhaustion, depersonalization, and personal accomplishment), and the common psychological symptoms of depression, anxiety, and stress; (2) employ EI and BEI metrics to identify the most influential nodes within physician burnout and other psychological symptoms network. Given previous research suggesting emotional exhaustion has a stronger association with depression than it with anxiety (27), we hypothesize that emotional exhaustion in physician burnout will exhibit the strongest positive correlation with depressive symptoms. Additionally, earlier studies have indicated that alleviating burnout symptoms, especially emotional exhaustion, effectively improves depressive symptoms. As a result, we propose that interventions targeting emotional exhaustion in physician burnout may act as bridge nodes within the psychological symptom cluster, opening new avenues for enhancing overall psychological well-being.

## 2 Methods

### 2.1 Study design and participants

The current study data were obtained from a larger research project, the 2021 National Hospital Performance Evaluation Survey (NHPES) which was endorsed by the National Health Care Commission of China. Conducted between January and March 2021, the objectives of NHPES were to enhance mental health services, improve medical care quality, optimize healthcare professionals’ working environment, and inform national medical policy and resource allocation., The survey recruited psychiatrists, nurses, psychologists, and pharmacists from tertiary care psychiatric hospitals nationwide in China. A total of 21,858 staff members completed the online questionnaire via the National Health Commission’s “Health China” WeChat account, out of which 3,973 were identified as psychiatrists. After excluding 190 based on predefined criteria (detailed in Supplementary materials), data from 3,783 responses were analyzed, yielding a 95.2% response rate. There were no significant differences in demographic factors, including gender (χ^2^ = 0.277, *p* = 0.599), marital status (χ^2^ = 4.041, *p* = 0.133), and educational achievement (χ^2^ = 2.04, *p* = 0.153), between the effective and ineffective groups of participants. The study was approved by the ethics committee (approval number: 202002-KYXM-02), and informed consent was obtained from all participants.

### 2.2 Measurement

#### 2.2.1 Demographic and work-related characteristics

Sociodemographic information of the participants were obtained via an author-designed questionnaire, including age, gender, marital status, and educational attainment. The questionnaire also assessed work-related factors associated with the COVID-19 pandemic by specifically inquiring about participants’ direct experience in managing COVID-19 cases. Before conducting this nationwide survey, we conducted a pilot study with approximately 300 healthcare professionals who self-identified as doctors, nurses, psychologists, and pharmacists to enhance the quality of this questionnaire. This selection of demographic and work-related factors aligns with the established research methodologies.

#### 2.2.2 Depression, anxiety, and stress scale-21

The Depression, Anxiety, and Stress Scale-21 (DASS-21), consisting of 21 items, is a widely utilized psychometric instrument for assessing symptoms of mental health conditions such as depression, anxiety, and stress (28). DASS-21 is extensively employed in diverse research investigations renowned for its simplicity and efficacy (29, 30). This scale contains three subscales to assess symptoms of: depression (items 3, 5, 10, 13, 16, 17, and 21); anxiety (items 2, 4, 7, 9, 15,19, and 20); and stress (items 1, 6, 8, 11, 12, 14, and 18). Participants rate each item on a four-point Likert scale ranging from “not applicable” to “very applicable.” The cut-off points for the three subscales are as follows: Depression subscale score not lower than 10, Anxiety subscale score not lower than 8, and Stress subscale score not lower than 8. The present study demonstrated a high level of internal consistency for the DASS-21 scale, as indicated by a Cronbach’s alpha coefficient of 0.951, which exceeds the widely accepted threshold value (≥0.7) (31). Furthermore, individual assessments of the subscales revealed excellent reliability, with the Depression subscale exhibiting a Cronbach’s alpha coefficient of 0.906 and the subscales of Anxiety and Stress showing coefficients of 0.854 and 0.888, respectively.

#### 2.2.3 Maslach burnout inventory-human service survey

In our study, the Maslach Burnout Inventory-Human Services Survey (MBI-HSS) (18) was employed to evaluate levels of physician burnout. This scale comprises 22 items, rated on a 7-point Likert scale (0–6), and covers three dimensions: emotional exhaustion (items 1, 2, 3, 6, 8, 13, 14, 16, and 20), depersonalization (items 5, 10, 11, 15, and 22), and personal accomplishment (items 4, 7, 9, 12, 17, 18, 19, and 21, with reverse scoring). Individuals who achieve a score of 27 or higher in emotional exhaustion or 10 or above in depersonalization are classified as experiencing “burnout” (32, 33). The Mandarin version of MBI-HSS, which has been extensively utilized and validated in previous studies (34, 35), demonstrated a high level of reliability with a Cronbach’s alpha coefficient of 0.833 for the current sample. Furthermore, the dimensions of emotional exhaustion, depersonalization, and personal accomplishment exhibited satisfactory internal consistency with Cronbach’s alpha values of 0.912, 0.751, and 0.899, respectively.

### 2.3 Data analysis

The data analysis for this study was conducted using the R programming language in the RStudio environment (version 4.3.2) (36). The network reconstruction was achieved by employing the EBICglasso function in conjunction with the Spearman correlation. The EBICglasso method, which is a component of the lasso package, computes a sparse Gaussian graphical model using graphical lasso (37), with the tuning parameter determined by the Extended Bayesian Information Criterion (EBIC). The proposed approach effectively reduced the number of edges in the network, thereby facilitating a more lucid and interpretable representation (38). Our analysis focused primarily on the EI index, which was selected due to its suitability in networks with negative weights and its recognition as a relevant centrality measure (39). Additionally, the BEI index was calculated to identify bridge symptoms using the bridge function via the R package networktools (40). We opted against employing alternative centrality metrics, such as closeness and betweenness, due to their limited effectiveness in uncovering psychological variables (41).

The network’s graphical representation was generated using the qgraph R software package (version 1.9.8) (38). In these visual models, nodes (circles) were interconnected by edges (lines), with the thickness of the edges representing the strength of interactions. Positive associations were depicted in dark green, while negative associations were shown in red. Nodes with strong associations were clustered together, and those with weaker associations were positioned on the periphery, utilizing the Fruchterman-Reingold force-directed algorithm (42). The *R*^2^ predictability Index, calculated using the mgm R package (1.2-14) (43), quantified the variance explained by each node in connection with other nodes in the network. This index was visually depicted by the size of a semi-circular area surrounding each node on the graph.

The bootnet R package (version 1.5.6) was employed to evaluate the accuracy and stability of network edges, conducting 1,000 bootstrap iterations (44). Edge accuracy was assessed by examining the 95% confidence interval (CI) of bootstrap edge weights, with narrower intervals indicating higher precision. We further evaluated the robustness of centrality measures by comparing the association between centrality indices obtained from the complete sample and those derived from a 70% reduced sample. The Centrality Stability coefficient (CS coefficient) was also computed to assess network robustness. A value of ≥0.5 indicates high reliability, 0.25 to 0.5 signifies moderate reliability, and < 0.25 reflects a less robust network (45).

## 3 Results

### 3.1 Demographic characteristics, burnout, and psychological symptoms

Table 1 presents the demographic data of the sample. Among the participating psychiatrists, 1,521 (40.2%) were male, and 2,262 (59.8%) were female. The median age was 38.0 years, with an interquartile range (IQR) of 12.0 years. Notably, approximately one quarter (25.1%) of the participants reported experiencing burnout symptoms (95% CI: 23.7–26.4%). Regarding psychological symptoms, 1,011 participants (26.7%) endorsed depressive symptoms (95% CI: 25.3–28.1%), 913 individuals (24.1%) reported anxiety symptoms (95% CI: 22.8–25.5%), and 416 people (11%) reported stress symptoms (95% CI: 10.0–12.0%).

**Table 1:** Demographic characteristics of 3,783 Chinese psychiatrists.

Variables	Median (IQR) or *N* (%)
Age (years)	38.0 (12.0)
Marital status	
Single	631 (16.7)
Married	3008 (79.5)
Divorced or widowed	144 (3.8)
Education level	
College/medical degree	2438 (64.4)
Master’s degree	1115 (29.5)
Doctoral degree	230 (6.1)
Frontline experience with COVID-19 patients	1019 (26.9)
Outcome measures	
Emotional exhaustion^a^	11.0 (15.0)
Depersonalization^a^	5.0 (7.0)
Personal accomplishment^a^	34.0 (17.0)
Depression^b^	4.0 (10.0)
Anxiety^b^	2.0 (6.0)
Stress^b^	6.0 (12.0)
Physician burnout^c^	948 (25.1)
Clinically relevant symptoms of depression^d^	1011 (26.7)
Clinically relevant symptoms of anxiety^e^	913 (24.1)
Clinically relevant symptoms of stress^f^	416 (11.0)

a MBI-HSS.

b DASS-21.

^c^ Emotional exhaustion (≥ 27) or depersonalization (≥ 10) scores.

d Depression overall-score ≥ 10.

e Anxiety overall-score ≥ 8.

f Stress overall-score ≥ 8.

### 3.2 Estimation of the network and centrality between burnout and psychological symptoms

**Figure 1:**
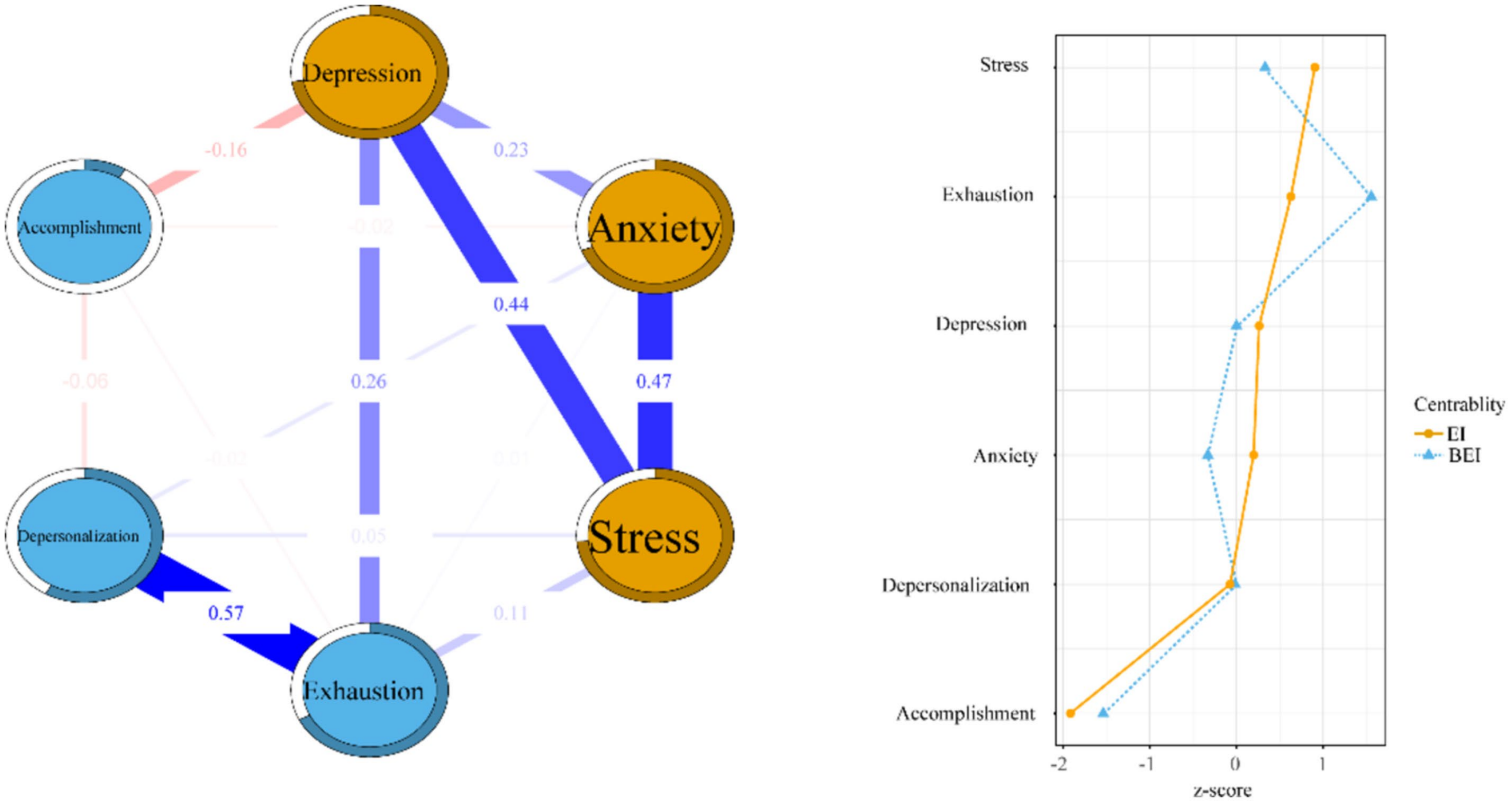
Estimation of network structure and centrality indices for all dimensions of DASS-21 and MBI-HSS.

The left side of Figure 1 illustrates the network structure encompassing physician burnout, depression, anxiety, and stress. Within this comprehensive network, 11 of the potential 15 connections (73.3%) exhibited nonzero values, highlighting significant interconnectivity among the symptoms. The average edge weight stood at 0.187, reflecting the overall robustness of the connections within the network. Predictability for individual nodes ranged from 8.7 to 73.5%, with an average of 58.3%. Among the symptoms, stress and depression had the highest predictability, while personal accomplishment had comparatively lower predictability in the burnout categories.

The strongest connection was observed between emotional exhaustion and depersonalization (*r* = 0.568). Weaker associations were found between anxiety and stress (*r* = 0.468), depression and stress (*r* = 0.437), depression and emotional exhaustion (*r* = 0.259), and depression and anxiety (*r* = 0.227).

### 3.3 Centrality and bridge symptom analysis

The left side of Figure 1 shows the EI and BEI within the network structure. Stress had the highest EI, indicating its dominant role in explaining the network model, followed by emotional exhaustion, depression, anxiety, and depersonalization. Personal accomplishment exerted minimal influence on the network’s structure.

Emotional exhaustion had the highest BEI, suggesting its critical role in connecting different symptom clusters. Among the inter-group connections, emotional exhaustion and depression had the strongest association, indicating a key link within the network.

### 3.4 Specific associations between burnout and psychological symptoms

**Figure 2:**
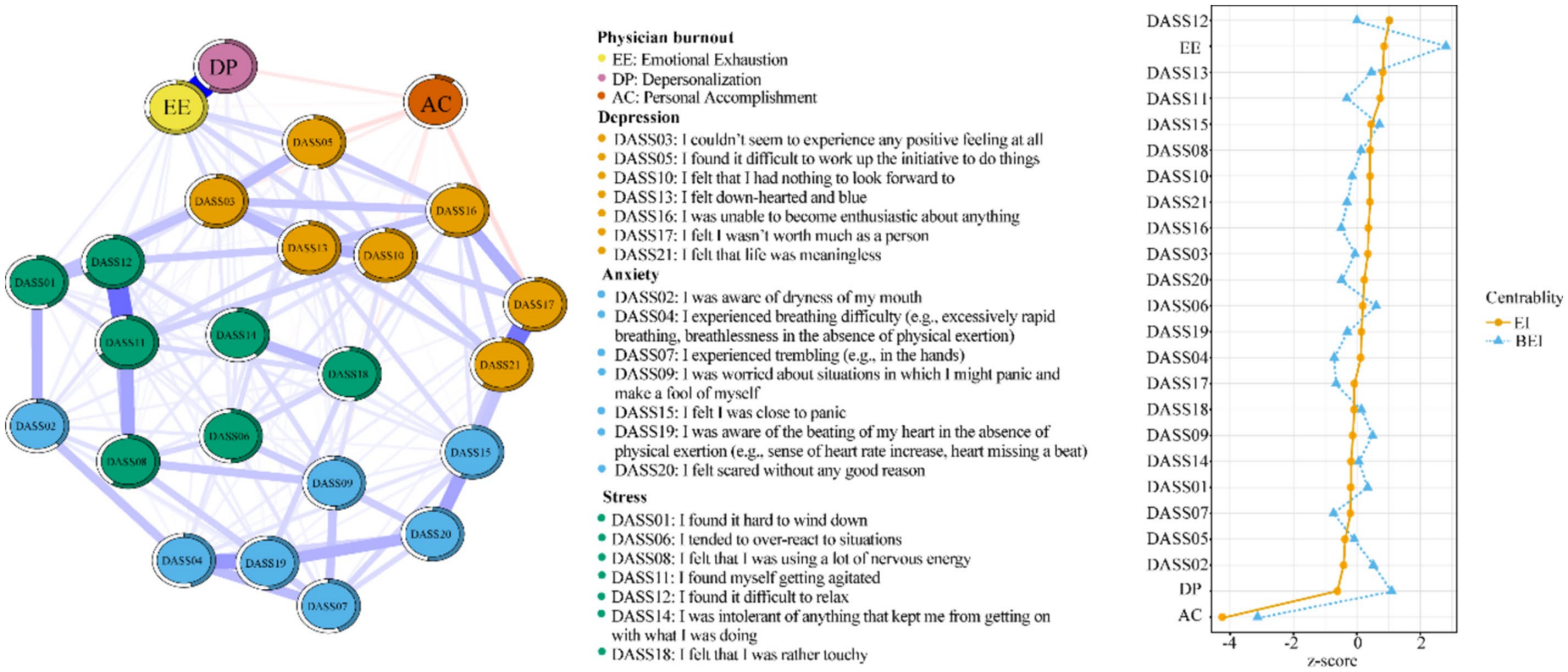
Estimation of network structure and centrality indices for all items of DASS-21 and MBI-HSS dimensions.

Figure 2 depicts the associations between the three dimensions of physician burnout and psychological symptoms. Emotional exhaustion was strongly correlated with DASS-21_10 (“I felt that I had nothing to look forward to”), DASS-21_05 (“I found it difficult to work up the initiative to do things”), and DASS-21_03 (“I could not seem to experience any positive feeling at all”). Depersonalization was linked to DASS-21_18 (“I felt that I was rather touchy”) and DASS-21_06 (“I tended to over-react to situations”). A reduced sense of personal accomplishment was associated with DASS-21_17 (“I felt I wasn’t worth much as a person”) and DASS-21_05 (“I found it difficult to work up the initiative to do things”).

### 3.5 Stability and precision of the network

**Figure 3:**
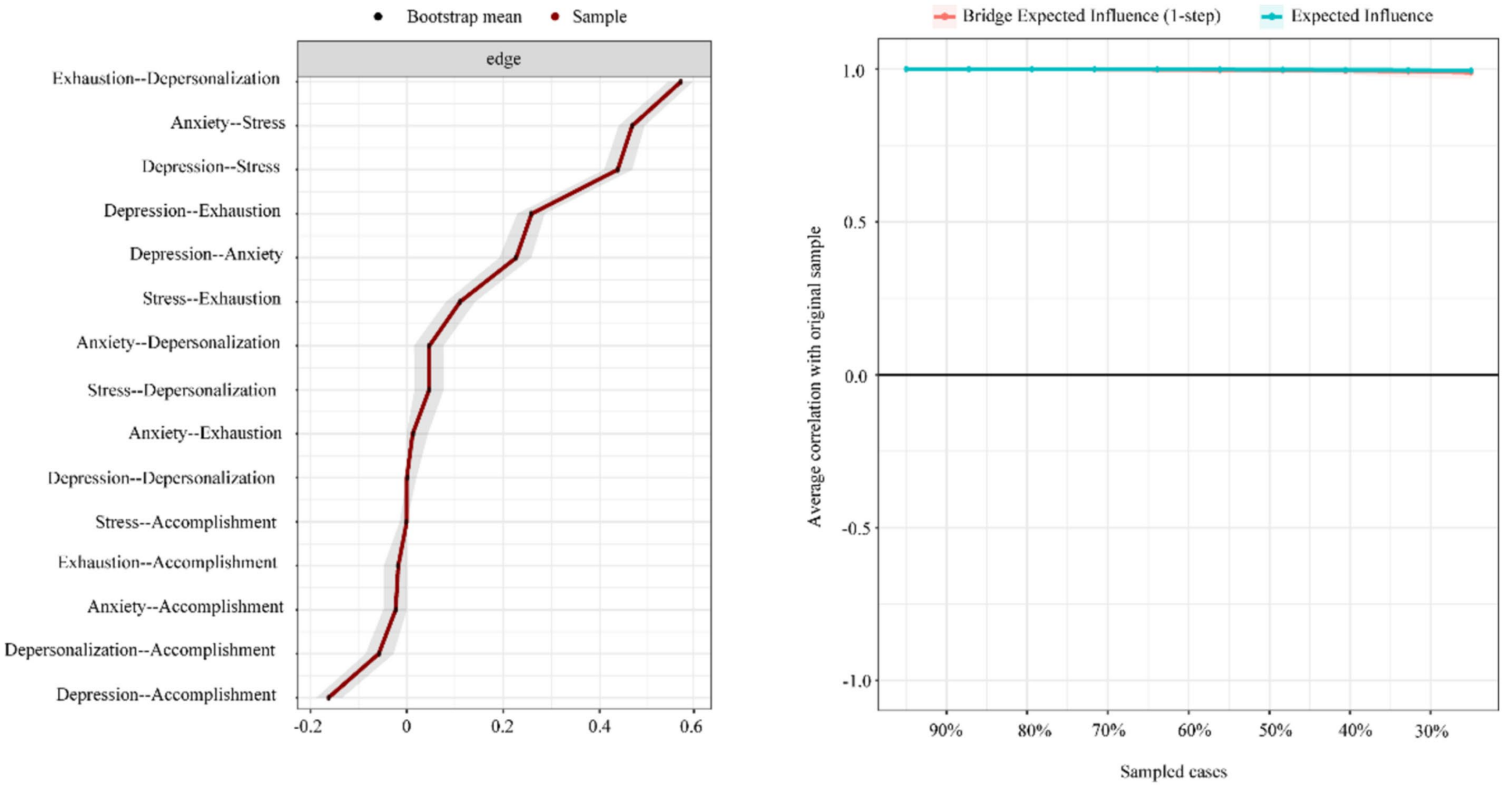
Network robustness and accuracy. Exhaustion, emotional exhaustion; Accomplishment, personal accomplishment.

To ensure the accuracy of edge measurements, the current study compared the average associations from resampling (bootstrap averages) with those obtained from the original sample. The left side of Figure 3 illustrates satisfactory accuracy, as evidenced by the convergence of the black and red lines. Additionally, the narrow gray band indicates minimal variability during resampling.

The stability analysis in the left side of Figure 3 robustly confirms the network model’s reliability, focusing particularly on the stability of edge weights. This demonstrates a dependable estimation of connection strength. Remarkably, even when excluding cases, there is a consistent observation of an average correlation between original and resampled data that significantly surpasses the 0.75 threshold, maintaining this high level. Both measures of EI and BEI affirmed our analysis’s reliability with stability coefficients (CS) of approximately 0.75.

## 4 Discussion

This study highlights the significant mental health challenges faced by Chinese psychiatrists, revealing that approximately 25.1% experienced symptoms of burnout. This finding is consistent with a meta-analysis by Bykov et al. (46), which reported an overall prevalence of physician burnout at 25.9% across 36 studies. Such alignment emphasizes that physician burnout is a widespread issue in healthcare systems across different cultures and institutions. However, unlike previous studies that mainly focus on prevalence rates, our study provides deeper insights by utilizing network analysis to explore the intricate relationships between different dimensions of burnout (emotional exhaustion, depersonalization, and personal accomplishment) and psychological symptoms such as depression, anxiety, and stress.

Around one in four Chinese psychiatrists also reported symptoms of depression and anxiety, a finding consistent with Sahebi et al. (47), who observed similar rates among internists. Although Yao et al. (5) reported similar prevalence figures, our network analysis revealed stronger associations between depression and emotional exhaustion, highlighting the critical importance of addressing emotional well-being in high-pressure environments, particularly during crises like the COVID-19 pandemic.

This study also revealed that approximately one in four Chinese psychiatrists reported symptoms of depression and anxiety, aligning with findings from a meta-analysis among internists, which showed prevalence rates of depression and anxiety at 24.83 and 24.94%, respectively (47). The 11% prevalence rate of stress symptoms among Chinese psychiatrists also mirrors findings among psychiatric residents in Bangladesh (48). It is important to note that different assessment tools can yield varying results, as demonstrated by a study that used a single-item measure for stress symptoms and reported a significantly higher prevalence rate of 35.3% (49).

These findings are consistent with prior literature emphasizing the prevalence of burnout and psychological symptoms among healthcare workers globally, especially during crises like the COVID-19 pandemic (50, 51). For example, Dalmasso et al. (52) evaluated the impact of individualized psychological support in the Bambino Gesù Paediatric Hospital in Rome and found that such interventions significantly improved healthcare workers’ mental health by reducing anxiety and stress levels. This suggests that similar psychological support interventions could be highly beneficial for Chinese psychiatrists, particularly in addressing the psychological distress highlighted in our study.

The dense regularized network offers a deep dive into the complex associations between the three dimensions of physician burnout—emotional exhaustion, depersonalization, and personal accomplishment—and the other mental health variables in this study –depression, anxiety, and stress, unveiling notable differences among them. Emotional exhaustion was strongly linked to multiple mental health symptoms within the DASS (Depression, Anxiety, and Stress Scales) community, particularly within the depression spectrum. This is evident in three key indicators: DASS-21_10 (“I felt that I had nothing to look forward to”), DASS-21_05 (“I found it difficult to work up the initiative to do things”), and DASS-21_03(“I could not seem to experience any positive feeling at all”). These connections highlight how closely emotional exhaustion is related to an individual’s motivation and the ability to experience joy and positivity. Previous research has indicated that feelings of despair and an absence of pleasure contribute to the exacerbation of emotional exhaustion (53, 54).

In previous network analyses, emotional exhaustion has also been identified as a key component of burnout, often serving as a central link between burnout and other psychological symptoms, such as depression and anxiety (22, 55). Our findings expand on this by identifying emotional exhaustion as a critical bridge symptom in the burnout network, suggesting that interventions targeting emotional exhaustion could help prevent the progression of burnout into more severe mental health issues. This is in line with research by Maslach and Leiter (22), who emphasized emotional exhaustion as one of the first indicators of burnout. Therefore, targeted interventions focusing on reducing emotional exhaustion could have far-reaching effects on preventing other mental health complications (56).

Depersonalization, characterized by an indifferent attitude towards work and feeling like an outside observer of oneself, was found to be correlated with DASS-21_18 (“I felt that I was rather touchy”) and DASS-21_06 (“I tended to over-react to situations”) within the stress community. These associations suggest that depersonalization may lead to an increased sensitivity and overreaction to everyday situations (57), where enhanced reactivity could heighten tensions in interpersonal relationships and exacerbate the symptoms of depersonalization.

The central role of stress in our network model aligns with previous findings, where stress was found to play a pivotal role in mental health deterioration, particularly under high-pressure conditions (58–60). Our findings further support the assertion that stress can serve as a precursor to psychological disorders, including depression, anxiety, and burnout. Previous studies have also shown that stress can predict depression over time (59). Given the high stress levels reported during the COVID-19 pandemic, these findings underscore the importance of early interventions to manage stress in healthcare professionals.

Additionally, stress was shown to have the highest EI (Expected Influence) index in our network, reflecting its centrality in the burnout-depression-anxiety-stress network. This finding is supported by the work of Mihić et al. (61), who found that stress played a core role in the depression-anxiety-stress network in healthcare workers. Similarly, previous research has found that relaxation techniques such as mindfulness and yoga can be effective in reducing stress and its associated symptoms (62, 63). Therefore, implementing effective intervention strategies such as these becomes crucial when stress symptoms appear.

This study also unveils the pivotal role of emotional exhaustion as a bridge symptom in the nexus of physician burnout and other mental health symptoms, including stress, depression, and anxiety. Bridge symptoms are vitally important in network analyses, as they act as essential conduits for the interaction of different mental health issues, potentially leading to comorbidity or exacerbation of existing conditions (55). Emotional exhaustion has been recognized as a core symptom of physician burnout in numerous studies (22). It often signals the commencement of physician burnout. By targeting bridge symptoms, clinicians can more effectively treat or prevent complications (56). This indicates that interventions designed to alleviate emotional exhaustion could have wider implications for promoting psychological well-being. Organization-based interventions, mindfulness-based stress reduction, and cognitive-behavioral therapy suggested by previous studies should be widely used in healthcare settings to mitigate emotional exhaustion (51, 64).

Our findings also highlighted emotional exhaustion and depersonalization had the strongest link. This further confirms that physician burnout is not merely a manifestation of depression (22). However, empirical findings showed that emotional exhaustion and depression had a stronger association when compared to the link between depersonalization and personal accomplishment (21). When facing this controversial debate, it seems more appropriate to consider the two core symptoms of physician burnout, emotional exhaustion and depersonalization, are separate from symptoms of depression. Physician burnout, stemming directly from prolonged work-related stress, affects not just an individual’s mental well-being but can also result in a detached approach to the workplace and responsibilities, or in other words, depersonalization. Additionally, while physician burnout and depression exhibit overlapping characteristics, physician burnout is intricately linked to the work environment, contrasting with depression, whose symptoms persist regardless of changes in the work setting (65).

Consistent with empirical findings, our research also confirmed that personal accomplishment had a minimal impact across the entire network, suggesting a limited association with the physician burnout-depression network dynamics. Emotional exhaustion (22) and depersonalization (66, 67) have been empirically considered as the primary domains of physician burnout measured by Maslach Burnout Inventory-Human Services (MBIHSS), which is also the statistical tool applied in this study to assess physician burnout (23). Additionally, a previous study indicated that more than half of the individuals in the high burnout group experienced at least a moderate level of personal accomplishment (20).

While the current study highlighted important findings, it is crucial to acknowledge its limitations. First, as a cross-sectional study, it does not capture the dynamic changes in burnout and psychological symptoms over time. This design limits our understanding of the long-term psychological impact. Future research should consider employing a longitudinal design to track changes in mental health and burnout over time, offering a more comprehensive understanding of the pandemic’s lasting effects on psychiatrists.

Second, this study did not include a control group of non-psychiatric healthcare professionals, making it difficult to differentiate the specific factors contributing to burnout among psychiatrists compared to other healthcare workers. Including a control group in future research would help to better identify the unique stressors faced by psychiatrists and provide more targeted intervention strategies.

Third, the study did not assess the use of psychotherapy or counseling interventions, which may serve as protective factors in alleviating burnout and associated symptoms. The absence of data on these interventions is a limitation, and future research should evaluate their potential role in preventing and mitigating mental health issues among psychiatrists.

Lastly, while this study underscores the need for psychological support, it did not assess the effectiveness of specific interventions. Future studies could focus on evaluating the impact of psychological support programs on reducing burnout and improving mental health outcomes for psychiatrists, especially in the context of the post-pandemic recovery.

To our knowledge, this is the first study to investigate the network of physician burnout and symptoms of depression, anxiety, and stress among Chinese psychiatrists during the COVID-19 pandemic. By employing innovative methodologies to quantify the strength and centrality of connections among various symptoms, this research offers a more holistic approach to comprehending mental health disorders and identifies highly centralized symptoms in networks, such as stress, which may shed light on targeted therapeutic interventions.

## Data Availability

The original contributions presented in the study are included in the article/Supplementary material, further inquiries can be directed to the corresponding authors.
