# Supplementary material for: Unraveling the interconnectedness between physician burnout and symptoms of depression, anxiety, and stress: a network analysis among Chinese psychiatrists
Source: Front Public Health. 2025 Jan 7;12:1493424. doi: 10.3389/fpubh.2024.1493424 (PMC11746075; doi:10.3389/fpubh.2024.1493424)
Supplement: Supplementary file 1 [file Data_Sheet_1.docx]

**Supplementary Appendix**

Table S1. Edge strength in the network of mental health symptoms and emotional exhaustion among psychiatrists.

Table S2. Edge strength in the network of mental health symptoms and depersonalization among psychiatrists.

Table S3. Edge strength in the network of mental health symptoms and personal accomplishment among psychiatrists.

Figure S1. Bootstrap edge weights difference test of the node’s strength in the network is shown in Figure 1.

Figure S2. Bootstrap edge weights difference test of the node’s strength in the network is shown in Figure 1.

Figure S3. Bootstrap edge weights difference test between edge weights in the network shown in Figure 3

Material 1. E-logical setting

**Table S1. Edge strength in the network of mental health symptoms and emotional exhaustion among psychiatrists.**

| **Edge** | **Partial correlation coefficient** | **Edge** | **Partial correlation coefficient** |
| --- | --- | --- | --- |
| DASS10 | 0.089 | DASS12 | 0.040 |
| DASS05 | 0.084 | DASS01 | 0.036 |
| DASS03 | 0.075 | DASS11 | 0.035 |
| DASS16 | 0.059 | DASS02 | 0.028 |
| DASS13 | 0.045 | DASS08 | 0.026 |
| DASS18 | 0.040 | DASS21 | 0.014 |

**Table S2. Edge strength in the network of mental health symptoms and depersonalization among psychiatrists.**

| **Edge** | **Partial correlation coefficient** | **Edge** | **Partial correlation coefficient** |
| --- | --- | --- | --- |
| DASS18 | 0.035 | DASS21 | 0.020 |
| DASS06 | 0.032 | DASS08 | 0.020 |
| DASS14 | 0.030 | DASS01 | 0.009 |
| DASS19 | 0.021 | DASS09 | 0.006 |

**Table S3. Edge strength in the network of mental health symptoms and personal accomplishment among psychiatrists.**

| **Edge** | **Partial correlation coefficient** | **Edge** | **Partial correlation coefficient** |
| --- | --- | --- | --- |
| DASS17 | -0.072 | DASS18 | -0.022 |
| DASS05 | -0.067 | DASS01 | -0.021 |
| DASS10 | -0.045 | DASS06 | -0.013 |
| DASS03 | -0.041 | DASS20 | -0.007 |
| DASS09 | -0.033 | DASS16 | -0.006 |


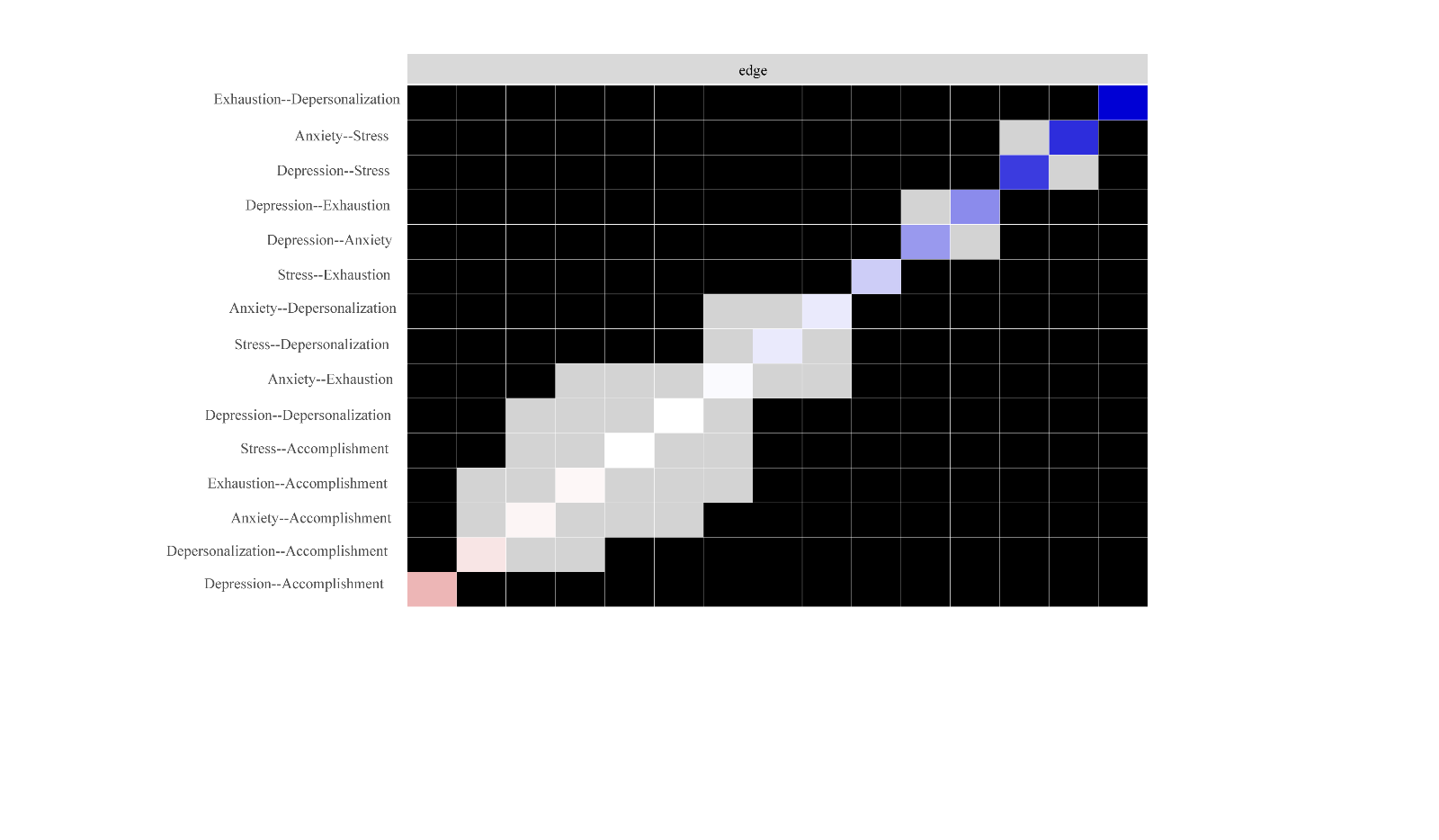
**Figure S1.** Bootstrap edge weights difference test of the node’s strength in the network is shown in Figure 1. *Note:* Black boxes represent significant differences in strength between two variables. Grey boxes indicate non-significant differences. The numerical magnitude of each node’s strength is indicated in the diagonal boxes.


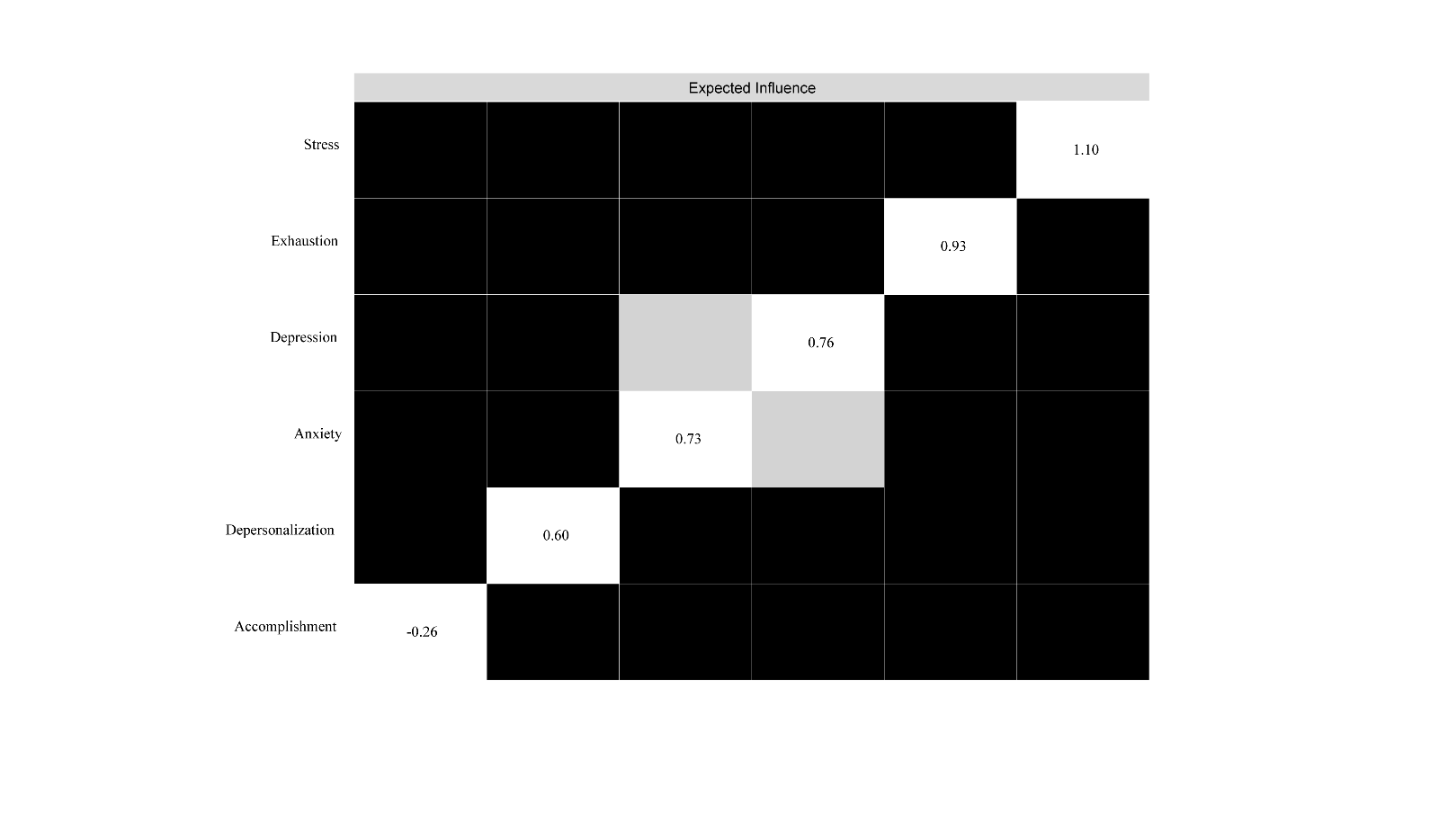


**Figure S2.** Bootstrap edge weights difference test of the node’s strength in the network is shown in Figure 1. *Note:* Black boxes represent significant differences in strength between two variables. Grey boxes indicate non-significant differences. The numerical magnitude of each node’s strength is indicated in the diagonal boxes.


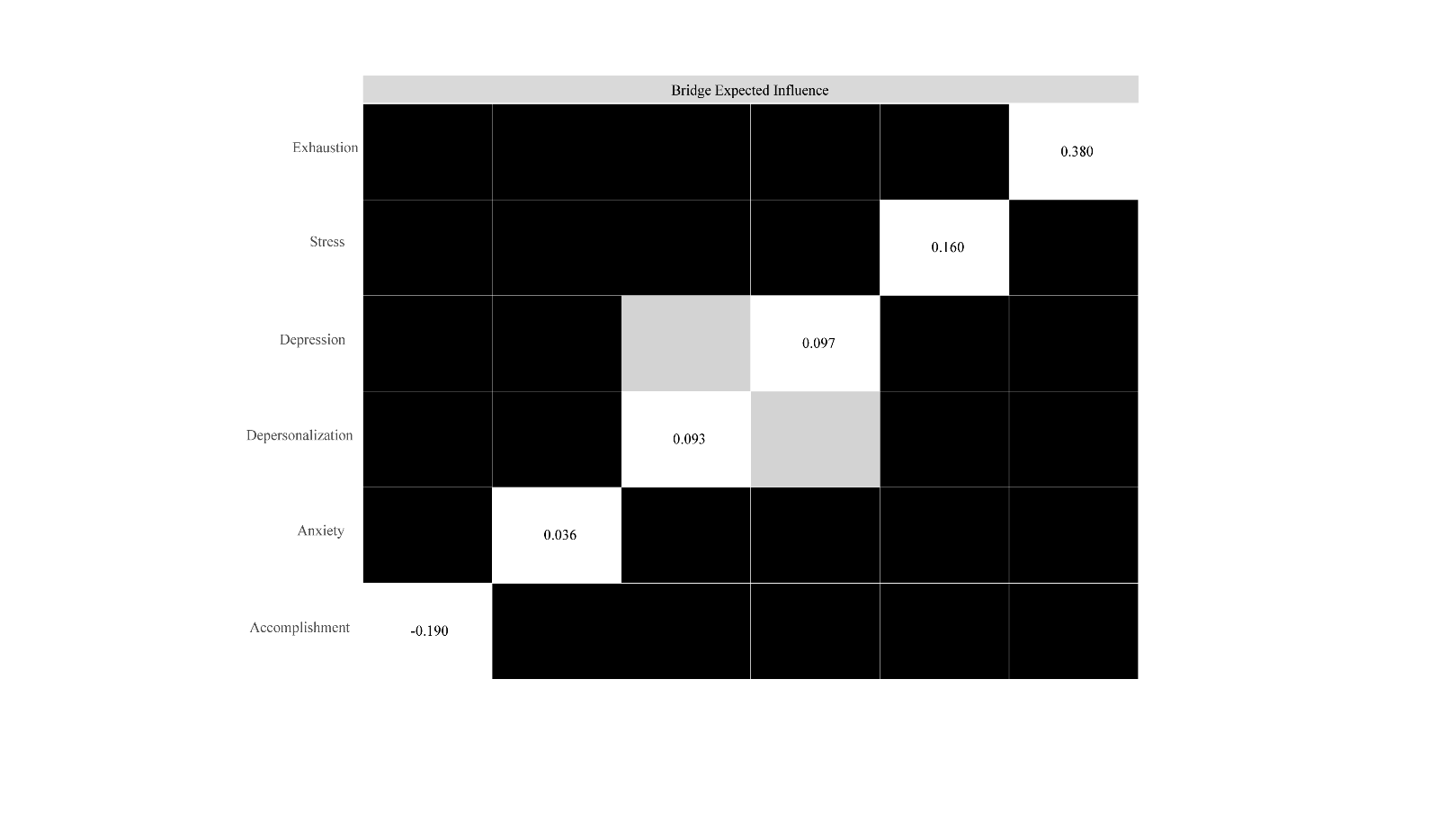


**Figure S3.** Bootstrap edge weights difference test between edge weights in the network shown in Figure 2

*Note:* Black boxes represent significant differences between nonzero estimated edges. Grey boxes indicate non-significant differences. The magnitude of each edge is characterized by the saturation of the color of the diagonal boxes (ranging from blue to red) corresponding to the magnitude of the edge (positive to negative)

**Material 1. E-****logical setting**

| **No** | **Logical setting** | **Number of participants excluded** |
| --- | --- | --- |
| 1 | Age minus years of service less than 18 years | 24 |
| 2 | Income less than 1000 or more than 100,000 RMB/month | 70 |
| 3 | Worked an average of <2 hours per day | 14 |
| 4 | Worked an average of >16 hours per day | 34 |
| 5 | Number of night shifts per month >15 | 12 |
| 6 | Received >500 outpatient visits per week | 36 |
| Total | | 190 (4.78%) |
